# Fabrication of Three-Dimensional Dendritic Ag Nanostructures: A SERS Substrate for Non-Invasive Detection

**DOI:** 10.3390/nano14191562

**Published:** 2024-09-27

**Authors:** Chia-Ling Sung, Tzung-Ta Kao, Yu-Cheng Lin

**Affiliations:** 1 Department of Engineering Science, National Cheng Kung University, Tainan 70101, Taiwan; n98131075@gs.ncku.edu.tw; 2 Institute of Photonics Engineering, National Kaohsiung University of Science and Technology, Kaohsiung 82445, Taiwan

**Keywords:** Ag nanodendrites, lactic acid, localized surface plasmon resonance (LSPR), surface-enhanced Raman scattering (SERS), SERS substrates

## Abstract

This paper discusses the fabrication of three-dimensional dendritic Ag nanostructures, showcasing pronounced Localized Surface Plasmon Resonance (LSPR) effects. These nanostructures, employed in surface-enhanced Raman scattering (SERS), function as sensors for lactic acid in artificial sweat. The dendritic structures of the silver nanoparticles (AgNPs) create an effective SERS substrate, with additional hotspots at branch junctures enhancing LSPR. We achieve differential LSPR effects by varying the distribution and spacing of branches and the overall morphology. Adjustments to electrodeposition parameters, such as current and plating solution protective agents on an anodized aluminum oxide (AAO) base, allow for precise control over LSPR intensities. By pre-depositing AgNPs, the electron transmission paths during electrodeposition are modified, which leads to optimized dendritic morphology and enhanced LSPR effects. Parameter optimization produces elongated rods with main and secondary branches, covered with uniformly sized, densely packed, non-overlapping spherical AgNPs. This configuration enhances the LSPR effect by generating additional hotspots beyond the branch tips. Fine-tuning the electrodeposition parameters improved the AgNPs’ morphology, achieving uniform particle distribution and optimal spacing. Compared to non-SERS substrates, our structure amplified the Raman signal for lactic acid detection by five orders of magnitude. This method can effectively tailor SERS substrates for specific analytes and laser-based detection.

## 1. Introduction

Raman scattering is a form of inelastic scattering that provides insights into molecular vibrations and structural properties. Its non-destructive nature enhances its value as a powerful tool in vibrational spectroscopy for material analysis. When monochromatic light interacts with a sample, most photons scatter elastically, retaining the same frequency, wavelength, and energy as the incident light. However, a small fraction of photons either absorb or lose energy, resulting in a frequency change known as inelastic scattering. The resulting shift in the position of the spectral signal is termed the Raman shift. This shift is associated with the vibrational modes of the molecules in the sample, providing detailed information about molecular composition and structure. By analyzing these Raman shifts, Raman spectroscopy generates spectra that serve as molecular fingerprints [1].

However, during Raman scattering measurements, the quantity of inelastic scattering produced after the illumination of the sample is exceedingly small, posing challenges to the measurement process. Surface-enhanced Raman scattering (SERS) technology overcomes the limitations of a weak signal and low sensitivity inherent in Raman spectroscopy while retaining the advantages of unique chemical fingerprint spectra and narrow-band peaks, thus gaining extensive application in the field of sensors. The discovery of SERS dates back to 1974, when M. Fleischmann et al. observed the Raman scattering spectrum signal of pyridine adsorbed on a roughened silver electrode surface [2].

SERS enables the highly sensitive structural detection of low-concentration analytes through the amplification of electromagnetic fields, which are generated by the excitation of Localized Surface Plasmon Resonance (LSPR). The enhancement mechanism of SERS, particularly when constructed from precious metals, is predominantly due to the electromagnetic (EM) effect. This EM effect is characterized by the collective oscillation of charge density, known as electric dipole oscillation, at the interface between the metal and the dielectric material, resulting in LSPR. These plasmonic hotspots (HSs), which are surface wave patterns polarized in the transverse magnetic (TM) mode, propagate along the interface created by the metal and dielectric substances. LSPR is particularly pronounced on the surfaces of metal nanoparticles (NPs), especially when their size is significantly smaller than the wavelength of the incident light. The conductive electron groups in metal NPs undergo electric dipole oscillation when influenced by an electric field. Moreover, LSPR is acutely sensitive to minute variations in factors such as the laser wavelength, nanostructure, size, shape, composition, and the interparticle spacing [3,4]. When the plasmon resonance wavelength (λ_LSPR_) equals half the sum of the excitation laser wavelength (λ_ext_) and the Raman scattering wavelength (λ_RS_), the SERS performance can reach its maximum. By adjusting the SERS structure to match different laser wavelengths, the plasmonic field can be maximized [5]. The λ_LSPR_ can be measured by spectroscopic instruments (UV-Vis) or obtained through calculations (Mie theory, finite-difference time-domain (FDTD) method), with the literature indicating that as the size of nanostructured silver decreases or the shape approaches a spherical one, a blue shift in the spectrum is observed [6,7,8].

SERS biosensors have the capability to detect a variety of biological samples, diseases, and explosives [3,9,10]. For instance, in research published by Hao Chen et al. in 2021, SERS technology was utilized to detect the COVID-19 virus. By employing a self-grown Au nanopopcorn surface, the SERS sensor was able to effectively detect and quantitatively analyze severe acute respiratory syndrome coronavirus 2 (SARS-CoV-2) lysates with high sensitivity within 15 min, achieving a limit of detection (LOD) of less than 10 PFU/mL [11]. Similarly, AgNPs functionalized with molybdenum disulfide (MoS₂) nanoflakes demonstrated excellent SERS performance, detecting pollutant molecules like methylene blue (MB) at ultra-low concentrations (as low as 5 nM), while also showing enhanced photocatalytic activity in the degradation of MB and rhodamine 6G (R6G) under sunlight [12]. Furthermore, MoS₂-Ag nanocomposite-based SERS substrates achieved ultra-low detection limits, with the incorporation of Ag significantly enhancing plasmonic effects for the highly sensitive detection of hazardous substances [13]. Additionally, gold (Au) NPs encapsulated within titanium dioxide (TiO₂) spheres have proven effective in tuning optical properties, leading to both improved photocatalytic activity and enhanced SERS detection, making them dual-function materials for environmental and biosensing applications [14].

Anodic aluminum oxide (AAO) is a self-assembled oxide film formed by anodizing the surface of aluminum. This oxide layer is characterized by its penetrating and densely packed hexagonal array of nanometer-scale columnar oxides, which are oriented perpendicular to the aluminum. Each columnar oxide within the array features a porous layer with a central hole, separated from the aluminum substrate by a thin yet dense barrier layer [15,16]. The self-assembled formation of this hexagonal pore array results from the repulsive forces generated between adjacent holes during the oxidation process, leading to the creation of an ordered AAO structure.

In contrast to other nanomaterials, AAO’s structure can be tailored to specific applications by controlling the size of its hexagonal nanopores and the thickness of the AAO film. This customization is achieved by adjusting various parameters, including the reaction temperature, electrolyte composition, and applied potential. Anodically oxidized aluminum is distinguished by its high chemical stability and insulating properties, coupled with a straightforward and cost-effective production process. Consequently, AAO finds extensive use across a broad spectrum of applications, such as in the synthesis of nanomaterials, electrical humidity sensors, optical sensors, photonic and electronic devices, photocatalysis, and various biological applications [17,18,19].

Silver nanostructures have garnered significant attention for their utility in chemical and biomedical sensing domains. For instance, Gao et al. achieved the detection of 5 M glucose using Ag nanodendrites on a Cu mesh substrate [20]. Similarly, Wang et al. successfully detected malachite green at a concentration of 9.4 × 10^−^^13^ M using Ag nanodendrites on an indium tin oxide (ITO) substrate [21]. In this research, three-dimensional Ag nanodendrites were synthesized via electrodeposition on AAO, subsequently integrating this structure into SERS applications. The electrodeposition process entails the reduction and deposition of silver ions onto AAO film, facilitated by the reaction between the electrolyte’s silver ions and the reducing agent. Electrochemical methods enable the low-energy preparation of SERS substrates with a controlled shape and size. Achieving a dendritic structure requires precise control over the current density and diffusion limitations. Furthermore, the morphology of Ag nanodendrites can be fine-tuned by adjusting various reaction conditions, including the electrolyte’s pH, concentration, temperature, and reaction duration.

During electrodeposition, alongside the oxidizing and reducing agents, a stabilizing agent known as polyvinylpyrrolidone (PVP, (C_6_H_9_NO)_n_) is introduced into the electrolyte. PVP, a water-soluble polymer, plays a pivotal role in stabilizing and regulating the size and shape of silver nanoparticles (AgNPs). It achieves this by enveloping the silver ions, thereby averting their agglomeration during the reduction process [22,23,24,25]. Upon exposure to incident light, the conductive electron groups within the Ag nanodendrites undergo intense collective oscillation due to the incident electric field’s influence. This distinctive morphology renders the Ag nanodendrites as highly effective SERS sensors.

In this paper, the SERS substrate is evaluated in comparison with a substrate that employs direct AAO electrodeposition. Prior to electrodeposition, AgNPs were deposited on anodized aluminum. Sodium borohydride (NaBH_4_) served as the reducing agent, while silver nitrate (AgNO_3_) facilitated the reduction of AgNPs through a chemical reaction. To prevent the agglomeration of AgNPs during the reaction, trisodium citrate (TSC, Na_3_C_6_H_5_O_7_) was utilized as a protective agent. Additionally, TSC promotes the formation of a spherical morphology in AgNPs. These AgNPs form the dendritic structure that guides the growth path during electrodeposition. This not only reduces resistance but also provides a discontinuous path for electron transport, resulting in a more granular (crystalline) surface on the electrodeposited Ag nanodendrites. By altering the characteristics of the Ag nanodendrites, LSPR of varying intensities can be achieved. This is accomplished by adjusting the dendrite plating parameters (current, plating solution protective agent) and modifying the electron transmission path during electrodeposition, which influences the morphology. The structural characterization is observed using scanning electron microscopy (SEM), and the variation in Raman signal intensity is employed to confirm the differences in the LSPR effect [7,26].

The quantitative detection of lactic acid (C_3_H_6_O_3_) is crucial, particularly since a significant increase in lactic acid production occurs when muscles lack sufficient oxygen during intense or strenuous exercise [27]. Lactic acid accumulation indicates the body’s inability to metabolize it efficiently, and monitoring blood lactic acid is considered a simple and effective prognostic indicator in surgical intensive care units. Po-Hsiang Hsu et al. reduced the detection limit of lactic acid from 50 mM using Raman spectroscopy to 0.01 mM by utilizing colloidal silver solutions [28]. Additionally, various disease factors can contribute to elevated levels of lactic acid. Compared to blood lactic acid detection, detecting lactic acid in sweat offers advantages such as being non-invasive, safe, and convenient. Typically, the concentration of lactic acid in sweat ranges from 4 to 25 mM, but it can surge to 50 mM or more during physical activity [29,30,31,32]. In this research, artificial sweat containing 4 mM lactic acid was employed as a detection sample to evaluate SERS signal intensity across different morphologies.

## 2. Materials and Methods

### 2.1. Chemicals

Sodium borohydride and silver nitrate were purchased from Acros. Lactic acid was obtained from Wako. Oxalic acid (C_2_H_2_O_4_·2H_2_O), phosphoric acid (H_3_PO_4_), TSC, urea (CH_4_N_2_O), copper (II) chloride (CuCl_2_), hydrochloric acid (HCl), sodium chloride (NaCl), and potassium chloride (KCl) were sourced from Miami Chemical. Sulfuric acid (H_2_SO_4_) was purchased from Choneye Pure Chemicals. PVP was obtained from Sigma-Aldrich. Deionized water (18.25 MΩ·cm) was used for all the prepared solutions.

The artificial sweat containing lactic acid was prepared according to the bubble method referenced in [33]. Lactic acid, urea, sodium chloride, and potassium chloride were added in proportion and diluted to a concentration of 4 mM.

### 2.2. Paperation of SERS Substrates

**Figure 1:**
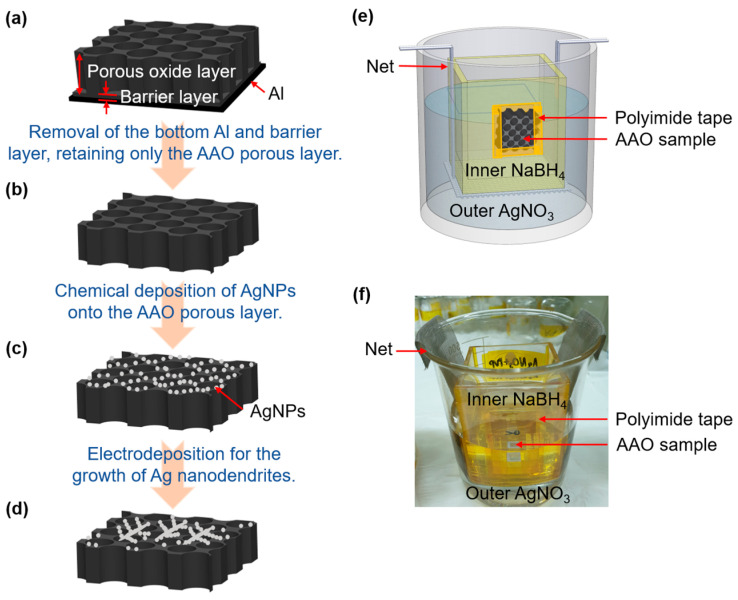
Process flowchart: (**a**) AAO, (**b**) porous layer of AAO, (**c**) AgNPs–AAO, (**d**) Ag nanodendrites–AAO, (**e**) a schematic diagram of the special container for depositing AgNPs, and (**f**) a photograph of the special container for depositing AgNPs.

The experimental procedure, depicted in Figure 1, involves the use of an oxalic acid and phosphoric acid solution as the electrolyte for anodization at a constant voltage of 160 V for 30 min. Subsequently, the same solution facilitates the voltage reduction process to achieve a thinner barrier layer (Figure 1a). Pre-thinning the barrier layer is a strategic step that significantly diminishes the risk of damage during its subsequent removal. To eliminate the underlying aluminum, a mixed solution of copper (II) chloride and hydrochloric acid is employed, followed by a phosphoric acid solution to dissolve the bottom barrier layer, ultimately isolating the AAO porous layer (Figure 1b). This porous layer serves as the foundation for chemical deposition utilizing silver nitrate, sodium borohydride, and TSC. The setup for deposition is showcased in Figure 1e,f. The solutions of silver nitrate, sodium borohydride, and TSC are applied to both sides of the AAO porous layer, where they diffuse through the pores and converge, following the solution ratios outlined in reference [6]. The resulting AgNPs are then deposited onto the surface of the AAO. This reaction proceeds for 2 h (Figure 1c). Utilizing the AgNPs as a conduit for electron transmission, a mixture of silver nitrate with either PVP or TSC is used to electrodeposit the Ag nanodendrites, with the process lasting 5 min (Figure 1d).

### 2.3. Instruments

The surface microstructure was observed using an ultrahigh-resolution scanning electron microscope (HR-SEM). This field emission electron microscope, the Hitachi SU-8000 model from Japan, operates within an accelerating voltage range of 0.5 to 30 kV.

**Figure 2:**
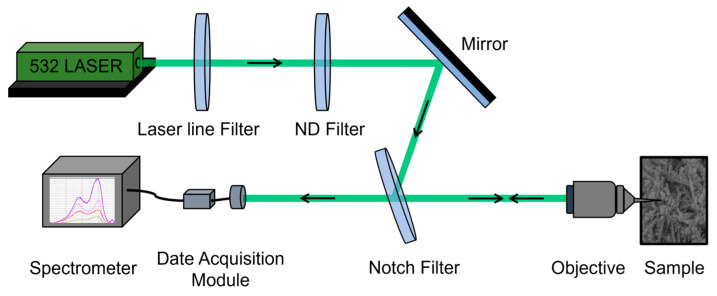
Schematic diagram of the Raman structure.

For Raman spectroscopy, a laser with a power output of 3 mW and a wavelength of 532 nm was employed. The Raman integration time, or exposure time, was set to 10 s. The configuration of the Raman setup is illustrated in Figure 2.

## 3. Results and Discussion

### 3.1. Characterization of AAO Base

An aluminum sheet was subjected to constant voltage anodization, where the nucleation of the oxide layer began from surface imperfections. The electric field intensifies in these depressions, accelerating the reaction rate and leading to the formation of hexagonal pores. Over time, a dynamic equilibrium is established between the dissolution of the pores and the growth of the underlying oxide layer. Subsequently, the voltage is decreased incrementally, disrupting this balance and focusing the electric field on the bottom barrier layer. This intensification accelerates the dissolution of the barrier layer, resulting in a thinner structure.

**Figure 3:**
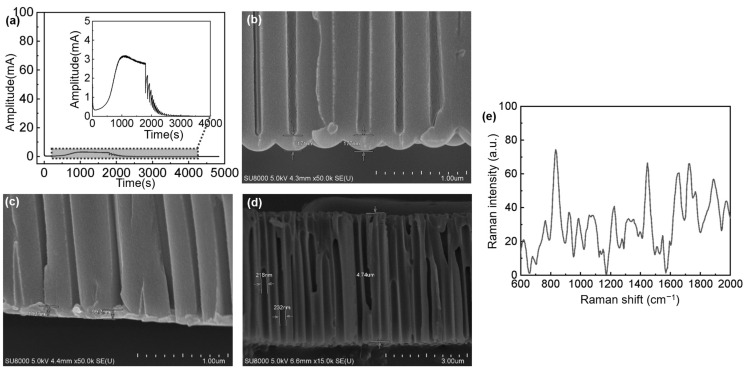
AAO: (**a**) voltage variation graph, (**b**) SEM cross-section without voltage reduction treatment, (**c**) SEM cross-section with voltage reduction treatment, (**d**) SEM cross-section of porous layer with the bottom barrier layer removed, and (**e**) Raman spectrum of 1 M artificial sweat measured on AAO.

The current and time dynamics of the anodization process are depicted in Figure 3a. Within the initial 10 s, the resistance increases due to the formation of the oxide layer, causing a sharp decline in current. As the oxide layer develops cracks and transitions into pores, the current begins to rise. The process stabilizes after about 30 min, with the current decreasing slowly as the bottom barrier layer grows and surface pores dissolve to a steady state.

At this juncture, the voltage reduction phase commences, with a gradual decrement of 4 V. This reduction concentrates the electric field at the base of the barrier layer. Following a slight rebound in the barrier layer, the voltage is further reduced by 4 V until no significant changes in current rebound are observed. The process concludes with a period of constant voltage growth at this reduced level for about 30 min, allowing the barrier layer to reestablish an appropriate thickness and pore density corresponding to the applied voltage.

Under typical constant-voltage anodic growth conditions, the barrier layer’s thickness is directly proportional to the applied voltage. However, since the AAO porous layer is the only component intended for retention, a voltage reduction step is implemented first to facilitate the subsequent removal of the barrier layer. A comparison between Figure 3b,c reveals that the barrier layer’s thickness without voltage reduction treatment is approximately 171 nm. Post voltage reduction, the barrier layer’s thickness is reduced to less than 113 nm. The process of thinning the barrier layer in the AAO minimizes the risk of damage when removing it, ensuring that only the porous layer remains, as illustrated in Figure 3d. Figure 3e shows the Raman spectrum for detecting 1 M lactic acid using the AAO base (non-SERS substrate). The figure indicates that, under the specified Raman conditions, the lactic acid signal is quite weak.

### 3.2. Characterization and Raman Signal of SERS Substrates

**Figure 4:**
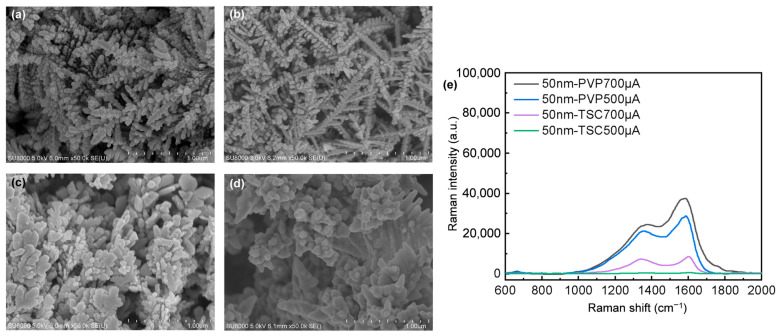
Images of 50 nm AgNPs: (**a**) PVP 500 μA, (**b**) PVP 700 μA, (**c**) TSC 500 μA, (**d**) TSC 700 μA, and (**e**) Raman spectrum of 4 mM artificial sweat measured on SERS substrates electrodeposited with different sizes of AgNPs.

Electroplating involves depositing 50 nm AgNPs on AAO. The protective agent in the electrodepositing solution serves a dual purpose: it prevents the clustering of AgNPs during the process and enables the creation of various morphologies by mixing different protective agents or adjusting their concentration. Ag nanodendrites are electrodeposited onto AAO, forming a sensitive SERS substrate (Figure 4). This process, guided by diffusion-limited aggregation (DLA) and Ostwald ripening, yields dense dendritic structures [34].

According to previously published papers, the deposition of AgNPs results in NP-connected nanodendrites, whereas without AgNPs, the nanodendrites exhibit a rod-like structure when electrodeposited on AAO [35]. The dendrites formed by the deposition of AgNPs enhance the LSPR effect. The presence of AgNPs during electrodeposition provides additional electron transport pathways, reducing resistance and, consequently, the potential [36]. Additionally, due to van der Waals forces, AgNPs adhere to the nanodendrite surface during the electrodeposition process [37]. These combined factors lead to a morphology characterized by NP-connected nanodendrites.

In this paper, PVP was utilized to electrodeposit nanostructures with distinct, slender main branches and branches, as depicted in Figure 4a,b. When the current was 500 μA, two distinct morphologies were observed. One exhibited slender main branches and branches, while the other displayed a seamless, feather-like sheet structure composed of closely packed AgNPs. At 700 μA, the structures predominantly showed slender main branches and secondary branches. The branches formed through TSC electrodeposition enveloped the main branches. At 500 μA, the branches exhibited an irregular flake morphology, whereas at 700 μA, an indiscernible dendritic structure of clustered AgNPs could be observed, as illustrated in Figure 4c,d.

The effect of LSPR on different SERS substrate morphologies was observed by detecting the Raman intensity of the SERS substrates using 4 mM artificial sweat (Figure 4e). It was observed that substrates with slender main branches and branches yielded a stronger signal compared to those with lamellar branches. This enhancement is attributed to LSPR, which benefits from the spacing and uniform spherical shapes of the branches that are closely arranged without overlap, resulting in a more pronounced LSPR effect.

However, the intensity of LSPR is influenced by multiple factors, including the overall morphology of the branches, as well as the number, spacing, and size of the AgNPs. It is important to note that higher currents do not necessarily equate to stronger LSPR, as branch formation is contingent upon current plating and diffusion limitations. The threshold for branch formation is also dependent on the materials, plating solutions, and other variables. This paper primarily focuses on how morphological changes can impact LSPR.

The LSPR response is amplified in structures characterized by prominent, elongated main branches and secondary branches decorated with AgNPs (Figure 4). Modifying the distribution of AgNPs along the electrodeposition pathway impacts electron transmission, leading to subtle morphological adjustments. Despite these changes appearing minimal, LSPR is highly sensitive to even minor variations in microstructure.

**Figure 5:**
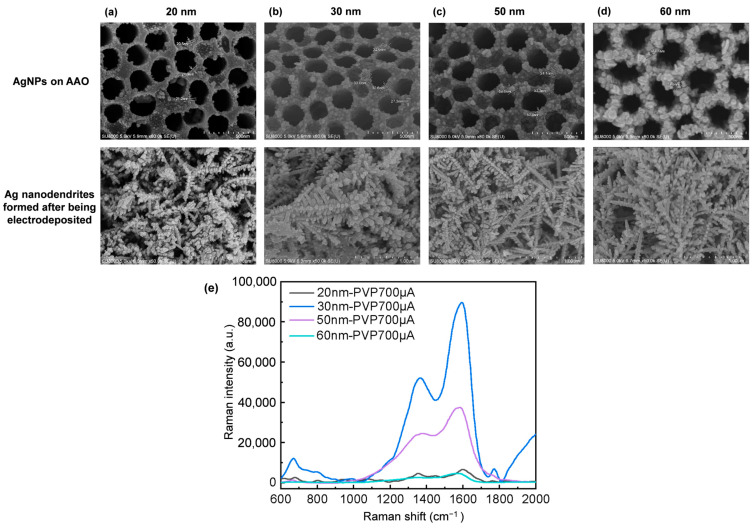
Corresponding Ag nanodendrites using different sizes of AgNPs: (**a**) 20 nm, (**b**) 30 nm, (**c**) 50 nm, (**d**) 60 nm, and (**e**) Raman spectrum of 4 mM artificial sweat measured on SERS substrates electroplated with different sizes of AgNPs.

During the electrodeposition process, the use of different AgNP sizes influences the growth pathways of branches, thereby altering the morphology (Figure 5). AgNPs with sizes of 20 nm, 30 nm, 50 nm, and 60 nm were individually employed as electron transfer pathways to compare their differences, with a consistent current of 700 μA. The resulting morphologies are characterized by slender main branches and secondary branches with attached AgNPs. Figure 5a shows structures deposited on AAO with a size of approximately 20 nm AgNPs. When Ag nanodendrites are electrodeposited on AAO using 20 nm AgNPs as electron transmission pathways, the AgNPs attached to the branches appear in high-density clusters with a predominantly spherical shape. In Figure 5b, when 30 nm AgNPs are used as electron transmission pathways, the AgNPs are evenly distributed along the branches, tightly arranged but without overlapping. In Figure 5c, it is shown that when 50 nm AgNPs are used as electron transmission pathways, compared to the 30 nm ones, there is only slight variation in the AgNPs attached to the branches, but the overall morphology is more uniform and consistent. Figure 5d shows that in the case of 60 nm AgNPs, the branches tend to cluster together, forming bush-like structures. Spherical structures of regular size and shape are known to enhance LSPR more effectively than rod-shaped counterparts. Additionally, the optimal NP size for LSPR varies with the laser wavelength used. The Raman signal intensity of Ag nanodendrite SERS substrates, electrodepositing AgNPs of different sizes as electron transmission pathways and detected with 4 mM artificial sweat, was observed to assess the LSPR effect (Figure 5e). The results confirm that AgNPs with a regular spherical shape on the branches lead to a better LSPR effect. Notably, in addition to the 30 nm AgNPs, the 50 nm AgNPs also exhibit superior signal performance. However, at a reduced current of 500 μA, as shown in Figure 4a, these larger AgNPs tend to cluster, forming feather-like structures. Therefore, the current settings for electrodeposition with 30 nm and 50 nm AgNPs will be adjusted to optimize the substrate’s LSPR response. Furthermore, it is speculated that the signal from Ag nanodendrites electrodeposited with 30 nm and 50 nm as electron transmission pathways is significantly higher than the other two sizes. This is likely due to the more uniform and extensive distribution of nanosilver particles deposited on AAO, as shown in Figure 5a–d of AgNPs on AAO.

**Figure 6:**
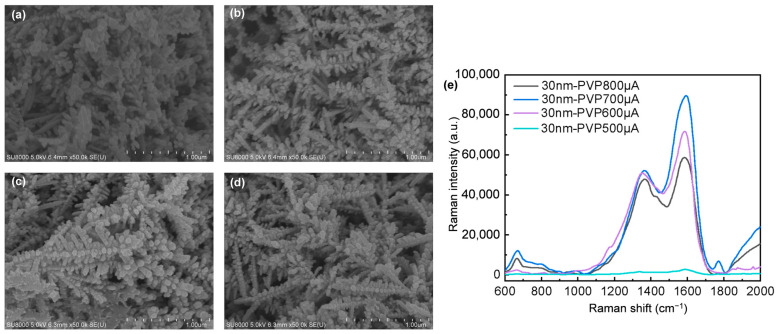
The electrodeposition of Ag nanodendrites under different currents is conducted using 30 nm AgNPs as electron transmission pathways, with PVP serving as the protective agent: (**a**) 500 μA, (**b**) 600 μA, (**c**) 700 μA, (**d**) 800 μA, and (**e**) Raman spectrum of 4 mM artificial sweat measured on SERS substrates electrodeposited with different currents.

The morphology of electrodeposited Ag nanodendrites is highly dependent on the current; inadequate current results in the inability to form dendritic shapes. However, minor adjustments in the current enable control over slight variations in dendritic structure. When utilizing 30 nm AgNPs as electron transmission pathways, modifying the electrodeposition current influences the dendrites’ structure (Figure 6). Despite the similarity in dendritic shapes, subtle adjustments in the gaps can amplify the Raman signal. Figure 6a shows that at 500 μA, the AgNPs on the branches exhibit irregular morphology with uneven sizes. As the current increases, the branches take on a spherical shape and become more uniformly arranged, as shown in Figure 6b–d (Figure 6c is the same as Figure 5b). Figure 6e illustrates the intensity variations when using 30 nm AgNPs as electron transmission pathways in Ag nanodendrites to detect 4 mM artificial sweat at different currents. This result indicates that slight variations in the spacing of AgNPs on the branches can impact LSPR. Additionally, according to previously published papers, SERS substrates fabricated using 30 nm AgNPs at 800 μA exhibit excellent reproducibility under consistent fabrication conditions. Furthermore, these substrates demonstrate remarkable stability across a range of artificial sweat concentrations from 0.25 mM to 4 mM [35].

**Figure 7:**
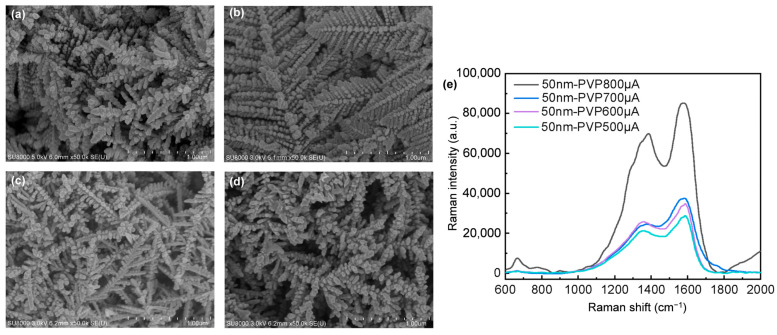
The electrodeposition of Ag nanodendrites under different currents is conducted using 50 nm AgNPs as electron transmission pathways, with PVP serving as the protective agent: (**a**) 500 μA, (**b**) 600 μA, (**c**) 700 μA, (**d**) 800 μA, and (**e**) Raman spectrum of 4 mM artificial sweat measured on SERS substrates electrodeposited with different currents.

Using a 50 nm electrodeposition pathway enables morphological variations through adjustments in the current (Figure 7). As indicated in Figure 7a (same as Figure 4a), under these conditions, two distinct morphologies emerge: one with slender main branches and AgNP-laden branches, and the other with a seamless, feather-like sheet structure of tightly packed AgNPs. Increasing the current from 500 μA to 600 μA transforms the slender branching into a more uniformly arranged feather structure, as shown in Figure 7b. Figure 7c (same as Figure 4b and Figure 5c) and Figure 7d show that when the current is set to 700 μA, the spacing between the AgNPs on the branches is relatively large. However, when the current is set at 800 μA, the AgNPs on the branches are closely spaced but do not overlap, which leads to a higher LSPR. Figure 7e shows the intensity variations when using 50 nm AgNPs as electron transmission pathways to detect 4 mM artificial sweat under different currents. The result indicates that Ag nanodendrites with regularly shaped spherical AgNPs and closely spaced but non-overlapping arrangements exhibit a better LSPR effect.

In the non-SERS substrate, the strongest band for lactic acid is observed at 830 cm^−1^, attributed to the stretching mode of the C–O bond within the carboxyl group (–COOH). However, on a SERS substrate, the characteristic peaks shift. Specifically, the band observed at 1400 cm^−1^, associated with the bending vibration of the carboxyl group (–COOH), is enhanced when lactic acid is adsorbed onto silver colloids. According to the literature, only the band at 1395 cm^−1^ provides a good signal-to-noise (S/N) ratio at low concentrations. The lactic acid signal in artificial sweat analyzed using SERS substrates is typically observed at 1400 cm^−1^ [38,39,40]. Under identical experimental conditions (laser wavelength, power, microscope objective, spectrometer, etc.), the amplification gain for artificial sweat was calculated using the analytical enhancement factor (AEF) formula [41]. The signal intensity of artificial sweat on the SERS substrate is denoted as I_SERS_ with concentration C_SERS_ and on the non-SERS substrate as I_RS_ with concentration C_RS_, as shown in Equation (1):

AEF = (I_SERS_/C_SERS_)/(I_RS_/C_RS_) (1)

As shown in Figure 3e, the intensity of 1M artificial sweat at 1400 cm^−1^ on the non-SERS substrate is 24.81, while the intensity of 4 mM artificial sweat on a SERS substrate can reach 67,212.43 (Figure 7e). Using Equation (1) for calculation, the Raman signal amplification gain for detecting lactic acid in artificial sweat is enhanced by five orders of magnitude.

## 4. Conclusions

This paper presents the successful fabrication of a three-dimensional dendritic silver nanostructure based on AAO, demonstrating its potential as a highly effective SERS sensor for detecting lactic acid concentrations in artificial sweat. The AAO base was chosen for its ability to create a uniform nanoporous structure over a large area, which significantly enhances the consistency and performance of the silver nanodendrites. 

The research indicates that extra hotspots favorable for LSPR are generated at the termini and intersections of the dendritic branches. By pre-depositing silver nanoparticles (AgNPs) on the AAO and adjusting electrodeposition parameters such as the current, protective agents in the plating solution, and the electron transmission path, we achieved precise control over the morphology of the dendritic structures. This approach led to optimized dendritic morphology and amplified LSPR effects, producing elongated rods with main and secondary branches covered with uniformly sized, densely packed, spherical AgNPs. The configuration of these nanostructures generates additional hotspots beyond the branch tips, which significantly enhances the LSPR effect.

The findings indicate that slender, straight branches are more effective at distributing hotspots compared to flake-like structures. The use of AgNPs of varying sizes (20 nm, 30 nm, 50 nm, and 60 nm) was found to influence the morphology, with all sizes capable of forming prominent dendritic structures. Adjusting the current enabled fine control over AgNPs’ morphology, leading to a more uniform distribution and optimal spacing between particles.

In applications for lactic acid detection in artificial sweat, the SERS substrate achieved a remarkable Raman signal amplification of five orders of magnitude compared to non-SERS substrates. Although AAO was not used as a variable in parameter modulation, its ability to adjust pore size and morphology offers potential for further optimizing SERS performance. This method effectively tailors SERS substrates for specific analytes and laser-based detection applications, paving the way for enhanced sensitivity and selectivity in sensor technologies.

## Data Availability

Data are contained within the article.
